# Cross-Modality Person Re-Identification via Local Paired Graph Attention Network

**DOI:** 10.3390/s23084011

**Published:** 2023-04-15

**Authors:** Jianglin Zhou, Qing Dong, Zhong Zhang, Shuang Liu, Tariq S. Durrani

**Affiliations:** 1 Tianjin Key Laboratory of Wireless Mobile Communications and Power Transmission, Tianjin Normal University, Tianjin 300387, China; 2 Department of Electronic and Electrical Engineering, University of Strathclyde, Glasgow G1 1QE, UK

**Keywords:** person ReID, graph attention network, cross-modality

## Abstract

Cross-modality person re-identification (ReID) aims at searching a pedestrian image of RGB modality from infrared (IR) pedestrian images and vice versa. Recently, some approaches have constructed a graph to learn the relevance of pedestrian images of distinct modalities to narrow the gap between IR modality and RGB modality, but they omit the correlation between IR image and RGB image pairs. In this paper, we propose a novel graph model called Local Paired Graph Attention Network (LPGAT). It uses the paired local features of pedestrian images from different modalities to build the nodes of the graph. For accurate propagation of information among the nodes of the graph, we propose a contextual attention coefficient that leverages distance information to regulate the process of updating the nodes of the graph. Furthermore, we put forward Cross-Center Contrastive Learning ($C^{3}L$) to constrain how far local features are from their heterogeneous centers, which is beneficial for learning the completed distance metric. We conduct experiments on the RegDB and SYSU-MM01 datasets to validate the feasibility of the proposed approach.

## 1. Introduction

The purpose of person re-identification (ReID) [1,2,3,4] is to match pedestrians across multiple non-overlapping cameras, which could be considered to be a specific person-retrieval task. It is extensively applied in smart cities, autonomous driving, security surveillance, and so on. However, most person ReID methods focus on matching pedestrians captured by RGB cameras, and therefore they do not allow 24-h intelligent surveillance. To overcome this limitation, some researchers are dedicated to cross-modality person ReID.

Cross-modality person ReID [5,6,7,8,9] retrieves RGB pedestrian images from infrared (IR) pedestrian images and vice versa. It not only inherits the challenges of unimodality person ReID, such as the variations in postures, illumination, and camera view, but also possesses a large discrepancy between IR modality and RGB modality. The modality discrepancy results in an unreliable match due to different color and appearance information of IR images and RGB images.

**Figure 1:**
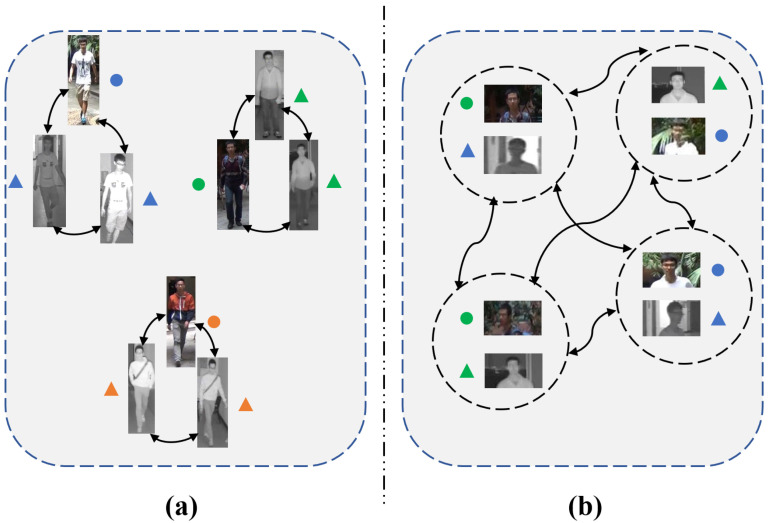
(**a**) Most methods for cross-modality person ReID treat each pedestrian image as a node of a graph. (**b**) The proposed method treats paired local features from different modalities as a node of a graph. We distinguish the pedestrian identities using different colors, with the same color indicating the same pedestrian. Circles and triangles are used to represent the IR and RGB modalities, respectively.

Recently, some cross-modality person ReID methods have been proposed to learn feature representations and metric functions for both IR and RGB images. Regarding feature representations, many methods [10,11,12] extract modality-specific features and modality-shared features by designing dual-stream deep networks. Meanwhile, the local features [13,14,15] are also demonstrated to be effective for cross-modality person ReID. Furthermore, some approaches [16,17,18,19] apply graph convolution layers, which aggregate features from other pedestrian images to enhance the discriminative power of features. They treat each pedestrian image as a node of a graph and update the nodes based on their correlations, as shown in Figure 1a. However, they ignore the relationship between the pairs of IR images and RGB images when constructing the graph, therefore hindering the learning of discriminative features for pedestrian images of different modalities.

In metric learning, some works [20,21,22] propose various losses to minimize the distance between pedestrian images from different modalities for cross-modal person ReID. These losses target the learning of an embedding space for different modalities in which images of same-identity pedestrians are closer to each other and images of different-identity pedestrians are further away. For this purpose, these methods constrain the distance among the pedestrian images from different modalities [23,24] or the distance among the centers from different modalities [25,26]. However, they overlook the distance between the pedestrian image and its center of different modalities, which results in incomplete distance learning between different modalities.

In this paper, we propose a novel graph network entitled Local Paired Graph Attention Network (LPGAT) for cross-modality person ReID. This approach considers the correlation of paired pedestrian images from different modalities and local information in a uniform framework. Specifically, we design the proposed method as a two-stream network where each branch corresponds to each modality. To learn local features, we uniformly divide the feature maps for each stream in the horizontal direction. Later, we construct a graph using local features where each node is composed of the corresponding local features of paired pedestrian images with varying modalities, which is illustrated in Figure 1b. The inclusion criterion of the selected paired local features is that each paired local features come from two different modalities. For better propagation of information in the graph, we further propose the contextual attention coefficient which not only considers the node features but also the relationship between the nodes. Hence, the proposed LPGAT could directly learn the relationship between the paired local features from different modalities and accurately propagate the information between them.

Recently, the distance constraint among the distinct modality centers has verified the effectiveness of cross-modality person ReID. However, they do not constrain the features which are far from the centers. To overcome this, we propose Cross-Center Contrastive Learning ($C^{3}L$) to reduce the distance between local features and their heterogeneous centers to narrow the gap between heterogeneous modalities. Combined with the constraint between heterogeneous centers, the proposed $C^{3}L$ helps learn the completed distance metric for IR images and RGB images, and therefore the discriminative features are obtained. The primary contributions are outlined below:

- We propose LPGAT for cross-modality ReID. In contrast to previous approaches that only use pedestrian images from different modalities as the nodes of a graph, LPGAT uses the paired local features from different modalities as the nodes of a graph, thus alleviating the gap between the two modalities.
- We propose $C^{3}L$ to constrain local features and their heterogeneous centers. In contrast to previous methods that only constrain the distance between the centers of different modalities, $C^{3}L$ constrains the features that are far from the center, thus narrowing the gap between heterogeneous modalities.
- We compare the proposed method against state-of-the-art methods using two publicly accessible datasets, RegDB and SYSU-MM01, and our results demonstrate that the proposed method outperforms them.

## 2. Related Work

### 2.1. Cross-Modality Person ReID

To address the challenge of the modality gap for cross-modality person ReID, many methods are proposed to derive global or local features from heterogeneous pedestrian images. For the global features, Wu et al. [27] put forward the deep zero-padding model to learn complementary information from IR images and RGB images. Then, Ye et al. [20] introduced a dual-stream network to capture the globally shared information of IR images and RGB images. Chen et al. [6] raised the Neural Feature Search (NFS) to implement the feature-selection automation, which allows the network to filter the background noise and focus on the important portions of pedestrian images.

Since pedestrians’ partial information is crucial for cross-modality person ReID, several researchers have proposed learning local features from IR images and RGB images. Zhu et al. [21] and Sun et al. [22] developed a deep two-stream framework to capture local features to mitigate modality differences. Zhang et al. [28] proposed Dual-Alignment Part-aware Representation (DAPR) to simultaneously reduce the modality gap and learn discriminant features from the local and global aspects. In this paper, we adopt a two-steam deep network to learn local features, and aggregate paired local features of pedestrian images from different modalities.

### 2.2. Graph Attention Networks

Graph Convolutional Network (GCN) [29,30] has been proposed to handle the non-Euclidean data. It learns node features by propagating the information among nodes as well as their neighborhoods. Several vision-related tasks, including semantic segmentation [31] and face analysis [32], have widely applied GCN. Later, Graph Attention Network (GAT) [33,34] is further proposed to aggregate node features using attention weights.

Recently, more and more researchers have combined GCN or GAT with Convolutional Neural Network (CNN) for person ReID [16,19,35]. Ye et al. [16] put forward Dynamic Dual Attention Aggregation (DDAG), in which each pedestrian image is regarded as a node of the graph, and the relationship between the node and its neighborhoods is mined. Zhang et al. [19] treated each body part as a node of a graph and construct a graph using one pedestrian image to alleviate the intra-modality variations. In this paper, a graph is constructed using paired local features derived from distinct modalities, and the contextual attention coefficients are introduced for better propagation.

### 2.3. Contrastive Learning

The purpose of contrastive learning [36,37] is to learn discriminative features using image pairs, so that similar images are close to each other, while dissimilar ones are far away. It can be applied in both unsupervised and supervised learning, such as image classification [37] and object detection [38]. Recently, contrastive learning has been used in person ReID to improve the discrimination of features [39,40]. For example, Chen et al. [39] proposed Inter-instance Contrastive Encoding (ICE) to fully explore the relationship between different pedestrian images. Isobe et al. [41] presented the Cluster-wise Contrastive Learning (CCL) algorithm to learn noise-robust features for cross-domain person ReID. We propose $C^{3}L$ to reduce the distance between local features and their heterogeneous centers to reduce the modality gap for cross-modality person ReID, which is inspired by the applications of contrastive learning in cross-domain ReID.

## 3. Approach

**Figure 2:**
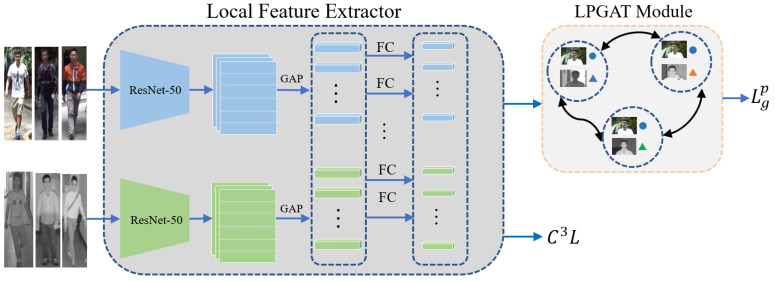
The framework of our approach. We first apply the Local Feature Extractor to obtain the local features from different modalities. Then, we propose the LPGAT module to learn the correlation between the paired local features from different modalities. The same color indicates the same pedestrian, and the circle and the triangle represent IR and RGB modalities, respectively. We also use the proposed $C^{3}L$ to optimize the network, which constrains the distance between the local features and their heterogeneous centers.

In this section, an outline of the proposed method is first represented, which is depicted in Figure 2. The proposed method contains three key components, namely Local Feature Extractor, LPGAT Module, and $C^{3}L$. Then, we introduce each of them in detail. Finally, we optimize the proposed approach.

### 3.1. Overview

**Local Feature Extractor.** The Local Feature Extractor is designed as a two-stream network where two individual ResNet-50 [42] are adopted as the backbone. Then, we divide the feature maps output by ResNet-50 horizontally and apply the global average pooling (GAP) to obtain the local features. Afterward, we apply the fully connected (FC) layers to reduce the dimension of local features, where the weights of FC layers are shared.

**LPGAT Module.** We regard the difference between paired local features of pedestrian images from different modalities as the node of a graph to learn the paired correlation of local features. Then, we update the nodes using the contextual attention coefficient which injects the distance information between the nodes into the process of information propagation.

**Cross-Center Contrastive Learning.** We introduce $C^{3}L$ to optimize the similarity between the local features and their heterogeneous centers. We then combine $C^{3}L$ with other metric functions to obtain the completed distance metric.

### 3.2. Local Feature Extractor

The Local Feature Extractor possesses two streams, and two individual pre-trained ResNet-50 are used as the backbone, where the stride of the convolution operation in the last layer is modified from 2 to 1. We feed the pedestrian images of IR modality and RGB modality into the two streams, respectively. Later, we obtain the feature maps of pedestrian images with the size of W×H×C, where *W* and *H* denote the width and the height of feature maps, and *C* is the number of channels. Afterward, we uniformly split the feature maps into *P* part-level stripes. We conduct GAP on each part-level stripe to obtain the local feature. The *p*-th local feature of the *i*-th RGB image is denoted as $f_{i,p}^{R}\in R^{C\times 1}$, where p=1,⋯,P. Similarly, the local feature of the IR image is denoted as $f_{j,p}^{I}\in R^{C\times 1}$. Finally, we apply the FC layers with shared weights to reduce the dimension of the local features from *C* to *D*.

### 3.3. LPGAT Module

The local features have proved the robustness to the variances in viewpoints, poses, and so on [1,13]. Meanwhile, the paired features derived from distinct modalities facilitate the reduction of the modality gap. Hence, we propose to use the paired local features from the IR modality and RGB modality to construct the fully connected graph. The node of the fully connected graph is defined as:

(1) $$g_{i,j}^{p}=f_{i,p}^{R}-f_{j,p}^{I}$$

Hence, we obtain a fully connected graph $G_{i}^{p}=\left\{g_{i,1}^{p},g_{i,2}^{p},\cdots ,g_{i,U}^{p}\right\}$ for the *p*-th local feature of the *i*-th RGB image, where *U* is the number of nodes in the graph. Please note that the node of a graph can also be performed by subtracting the local feature of RGB modality from the local feature of IR modality.

After obtaining the graph, we need to calculate the attention coefficient to describe the correlation between different nodes. Many cross-modality person ReID approaches [16,17] calculate the attention coefficient between the nodes as:

(2) $$\alpha_{n,m}^{p}=\frac{exp(\ell \left(q^{⊤}\left\lceil g_{i,n}^{p},g_{i,m}^{p}\right\rfloor \right))}{\sum_{u=1}^{U}exp\left(\ell \left(q^{⊤}\left\lceil g_{i,n}^{p},g_{i,u}^{p}\right\rfloor \right)\right)}$$

where *ℓ* is a nonlinear operation performed by LeakyReLU, ⌈,⌋ is the concatenation operation, and $q\in R^{2D\times 1}$ is a learnable vector.

From Equation (2) we can see that it directly concatenates the nodes, but ignores the relationship between the nodes, which leads to inaccurate information propagation. Thus, we propose the contextual attention coefficient:

(3) $$\alpha_{n,m}^{p}=\frac{exp(\ell \left(q^{⊤}\left\lceil g_{i,n}^{p},g_{i,m}^{p}\right\rfloor \right))}{\sum_{u=1}^{U}exp\left(\ell \left(q^{⊤}\left\lceil g_{i,n}^{p},g_{i,u}^{p}\right\rfloor \right)\right)}\times k_{n,m}^{p}$$

(4) $$k_{n,m}^{p}=exp(-∥g_{i,n}^{p}-g_{i,m}^{p}{∥}_{2})+\beta$$

where ${∥\cdot ∥}_{2}$ indicates the Euclidean distance, and β is the hyperparameter. From Equation (4), we can see that the smaller distance between the nodes possesses a larger $k_{n,m}^{p}$, and therefore it produces a strong correlation between the nodes. Hence, the contextual attention coefficient is helpful for accurate information propagation. Please note that when we set $k_{n,m}^{p}$ to 1, the contextual attention coefficient degenerates to the traditional attention coefficient. With the contextual attention coefficient, the node is represented as:

(5) $${\hat{g}}_{i,n}^{p}=\sum_{m=1}^{U}\alpha_{n,m}^{p}g_{i,m}^{p}$$

Finally, to further improve the representation ability, the node is updated as:

(6) $$e_{i,n}^{p}=\phi \left(w^{⊤}\left\lceil {\hat{g}}_{i,n}^{p},g_{i,n}^{p}\right\rfloor \right)$$

where ϕ is the ELU activation function to learn a stable graph structure, and $w\in R^{2D\times 2}$ is a learnable matrix. We treat the optimization of LPGAT as a binary classification problem, and use the verification loss:

(7) $$L_{g}^{p}=-\sum_{j=1}^{U}z_{i,j}^{p}\log{\bar{z}}_{i,j}^{p}-\left(1-z_{i,j}^{p}\right)\log\left(1-{\bar{z}}_{i,j}^{p}\right)$$

where ${\bar{z}}_{i,j}^{p}$ is the predicted probability of the *j*-th node of $G_{i}^{p}$, and $z_{i,j}^{p}$ is the ground-truth of the *j*-th node of $G_{i}^{p}$. $z_{i,j}^{p}=1$ indicates the paired local features derived from distinct modalities in the node are with the same identity, otherwise $z_{i,j}^{p}=0$.

In a word, we design the node of the graph in LPGAT as the paired local features derived from distinct modalities to effectively mitigate the discrepancy between IR modality and RGB modality. Furthermore, we inject the distance information using the contextual attention coefficient to propagate the information between the nodes accurately.

### 3.4. Cross-Center Contrastive Learning

**Figure 3:**
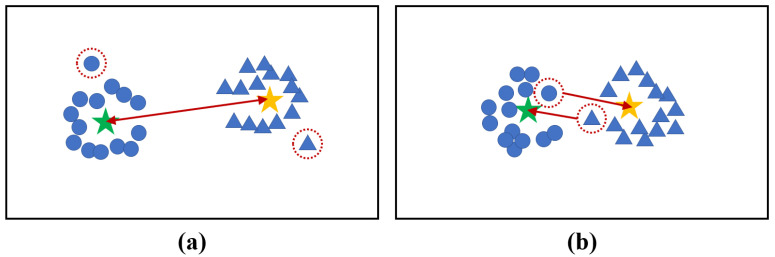
(**a**) The constraint on the centers of RGB modality and IR modality. However, there are some outliers in the learning process. (**b**) The proposed $C^{3}L$ constrains the local features and their heterogeneous centers, which alleviates the influence of outliers. The red arrows denote the constraints between features, whereas the dotted circles signify outliers. The stars indicate the centers, and the circle and the triangle represent the RGB modality and IR modality, respectively.

As for cross-modality person ReID, learning the distance metric is an effective way to narrow the modality gap. Recently, the constraint on the centers of RGB modality and IR modality have achieved promising performance [21,25,26]. However, they overlook the distance between features and their heterogeneous centers resulting in some outliers in the learning process as shown in Figure 3a.

In this paper, we put forward $C^{3}L$ to force the local features to be close to the corresponding heterogeneous centers in the embedding space, and therefore the pedestrian images which have the same identity from distinct modalities are gathered as shown in Figure 3b.

The constraint between the *p*-th local feature of the *i*-th RGB image and its heterogeneous center is defined as:

(8) $$L_{R}^{p}=-\log\frac{exp\{{\left(f_{i,p}^{R}\right)}^{⊤}c_{b,p}^{I}/\tau \}}{\sum_{s=1}^{S}exp\left\{{\left(f_{i,p}^{R}\right)}^{⊤}c_{s,p}^{I}/\tau \right\}}\;s.t.\;ID\left(i,V\right)=b$$

where τ>0 is a scalar temperature parameter, *S* is the number of identities, and ID(i,V) indicates the identity of the *i*-th RGB image. Here, $c_{b,p}^{I}$ is the center of the *p*-th local feature of the *b*-th identity for IR images, and it is defined as:

(9) $$c_{b,p}^{I}=\frac{1}{O_{b}}\sum_{i=1}^{O_{b}}f_{i,p}^{I}\;s.t.\;ID\left(i,I\right)=b$$

where $O_{b}$ is the number of pedestrian images with the *b*-th identity, and ID(i,I) is the identity of the *i*-th IR image.

Similarly, the constraint between the *p*-th local feature of the *i*-th IR image and its heterogeneous center is defined as:

(10) $$L_{I}^{p}=-\log\frac{exp\{{\left(f_{i,p}^{I}\right)}^{⊤}c_{b,p}^{V}/\tau \}}{\sum_{s=1}^{S}exp\left\{{\left(f_{i,p}^{I}\right)}^{⊤}c_{s,p}^{V}/\tau \right\}}\;s.t.\;ID\left(i,I\right)=b$$

where $c_{b,p}^{V}$ is the heterogeneous center of $f_{i,p}^{I}$. In a word, the proposed $C^{3}L$ is formulated as:

(11) $$L_{C}^{p}=L_{R}^{p}+L_{I}^{p}$$

The proposed $C^{3}L$ decreases the distance between the local features of IR images and their centers of RGB images, and so do the local features of IR images and their centers of RGB images. Hence, it clusters the local features of pedestrian images from different modalities with the same identity.

### 3.5. Optimization

For learning the completed distance metric, we exploit the proposed $C^{3}L$ as well as the heterogeneous center (HC) loss [21]. The HC loss aims to diminish the distance between the centers of IR modality and RGB modality with the same identity. The HC loss for the *p*-th local feature is denoted as $L_{HC}^{p}$. Additionally, we employ the cross-entropy loss to optimize the local features, and the cross-entropy loss for the *p*-th local feature is denoted as $L_{CE}^{p}$. Moreover, we use the validation loss $L_{g}^{p}$ to optimize LPGAT and treat it as a binary classification task. Therefore, the overall loss of the proposed method is expressed as:

(12) $$L=\frac{1}{P}\sum_{p=1}^{P}\left(L_{CE}^{p}+\lambda_{1}L_{HC}^{p}+\lambda_{2}L_{g}^{p}+\lambda_{3}L_{C}^{p}\right)$$

where $\lambda_{1}$, $\lambda_{2}$ and $\lambda_{3}$ are the trade-off parameters to balance the importance between different losses.

## 4. Experiments

In this section, the evaluation protocol and datasets are first presented, followed by showing the implementation details of our experiments. After that, the experimental results are compared with the state-of-the-art methods, and ablation experiments are performed to evaluate the effectiveness of the key components of the presented approaches. Finally, the influence of several important parameters in the proposed method is analyzed.

### 4.1. Datasets

We perform trials on the SYSU-MM0 [27] and RegDB [43], which are two public cross-modal datasets.

**SYSU-MM01,** a massive dataset, is captured by four visible-light cameras as well as two NIR cameras in both outdoor and indoor settings. There are 491 pedestrian identities recorded in this dataset, and each pedestrian is photographed by two different cameras. Furthermore, 11,909 IR images and 22,258 RGB images of 395 identities are contained in the training set. During the testing phase, we perform our experiments on two settings, i.e., indoor search and all search. Each mode has 3803 query IR pedestrian images of 96 identities. Additionally, the gallery set for all-search settings contains 301 randomly selected pedestrian images taken by RGB cameras which are placed in outdoor and indoor environments, while the gallery set for the indoor search settings contains 112 randomly selected pedestrian images taken by RGB cameras which are placed in indoor environments.

**RegDB** consists of 8240 images from 412 identities, with each identity containing 10 IR images and 10 RGB images. The whole dataset is divided into two halves and used for training and testing, respectively, which the training set includes 2060 IR images and 2060 RGB images of 206 identities. As for the test set, there are 2060 query images of 206 identities and 2060 gallery images of 206 identities. Moreover, two evaluation settings are available, including Thermal-to-Visible (T-V) and Visible-to-Thermal (V-T).

### 4.2. Evaluation Metrics

The Cumulative Matching Characteristic (CMC) curve is a commonly used performance evaluation metric in the person re-identification task. It plots the probability of correctly matching the query image at different ranks. Specifically, the *x*-axis represents the rank of the retrieved image (i.e., 1st, 2nd, 3rd, etc.), and the *y*-axis represents the probability of correctly identifying the query image among the top *k* retrieved images. A larger area under the CMC curve indicates better performance. The mean Average Precision (mAP) is another commonly used performance evaluation metric in the person re-identification task. It measures the average precision of a set of queries. Specifically, it considers both the precision and recall of the retrieval results. A higher mAP value indicates the better performance.

In this paper, standard CMC and mAP are adopted as evaluation metrics to test the performance of the proposed method.

### 4.3. Implementation Details

We first resize the pedestrian image into 288×144, then apply random cropping and shuffle a horizontal flip to augment the data. Meanwhile, we set the batch size to 64, where each batch is composed of 4 identities, and each identity consists of 8 RGB pedestrian images and 8 IR pedestrian images. The scalar temperature parameter τ in Equations (8) and (10) is set to 0.2. The hyperparameter β in Equation (4) is set to 2. To balance the importance between different losses, we set the trade-off parameters $\lambda_{1}$, $\lambda_{2}$, and $\lambda_{3}$ to 0.5, 0.4, and 0.5, respectively. We use stochastic gradient descent (SGD) optimizer to optimize the proposed method and fix the number of epochs to 60. The preliminary learning rate is set to 0.01 and decayed to 0.001 after 30 epochs. After that, We adopt the FC layers to reduce the local feature dimension to D=512, and the number of part-level stripes *P* is set to 6. In the testing phase, all local features are concatenated as the representation of a pedestrian image.

### 4.4. Comparisons with State-of-the-Art Methods

Table 1 and Table 2 show the results of the proposed methods on RegDB and SYSU-MM01 compared with the state-of-the-art methods, respectively. The compared methods mainly include the two-stream models (MSR [44], TONE [45], AGW [46]), GAN-based approaches (cmGAN [47], AlignGAN [48], JSIA-ReID [49]), modality aligning approaches (CMAlign [7]), and the metric learning (HCML [45], HSME [50]). In addition, some approaches learn the local features and meanwhile use the constraints between the heterogeneous centers (TSLFN+HC [21], WIT [22]).

**Comparisons on SYSU-MM01.** From Table 1, we can see that the proposed method achieves 61.89% of Rank-1 accuracy and 60.12% of mAP accuracy among the all-search setting, which exceeds NFS [6] and CMAlign [7] in terms of mAP accuracy by 4.67% and 5.98%, respectively. It is worth noting that the performance of our method exceeds that of DDAG [16] with respect to Rank-1 and mAP accuracy by 13.0% and 13.4%, respectively. This is because DDAG only uses pedestrian images of different modalities as the nodes of a graph, while our method uses the paired local features as the nodes of a graph. Compared with KSD [51], our method outperforms its Rank-1 and mAP accuracy by 1.3% and 2.3%, respectively. In addition, under the indoor search settings, the performance of our method surpasses that of WIT [22] by 5.8% and 5.9% regarding Rank-1 and mAP accuracy, respectively. This is because WIT uses the center constraint to pull images with the same identity to their cross-modality centers, but ignores constraining the features that are far from the centers. The proposed $C^{3}L$ overcomes this shortcoming by constraining the distance between local features and their heterogeneous centers.

The proposed method models the node of a graph with paired local features from different modalities, which outperforms other GAT models, such as DDAG. Furthermore, our approach yields superior performance to the other center-constrained approaches, i.e., TSLFN+HC [21] and WIT [22] on the indoor and all-search settings.

**Comparisons on RegDB**. We compare our LPGAT model with 13 different methods on the RegDB dataset. From the experimental results in Table 2, LPGAT shows the best performance compared with the other methods. Specifically, The proposed method obtains 89.37% in Rank-1 accuracy and 78.74% in mAP accuracy under the V-T mode, which surpasses the second-best method, i.e., WIT [22] with 4.37% and 2.84% in Rank-1 accuracy and mAP accuracy, respectively. In addition, under the T-V model, our method outperforms DDAG [16] and NFS [6] by 24% and 8.4% in Rank-1 accuracy, respectively, and surpasses them by 19.3% and 5.7% in mAP accuracy. Hence, it proves that our model has a strong generalization ability with different scenarios.

In conclusion, the proposed method yields superior performance on the two large-scale datasets, which demonstrates the good generalization capability of our approach.

**Table 1:** Comparisons with the state-of-the-art methods on SYSU-MM01 with two different settings. R-i indicates Rank-i. The bold indicates the best result.

Setting	All Search				Indoor Search			
**Method**	**R-1**	**R-10**	**R-20**	**mAP**	**R-1**	**R-10**	**R-20**	**mAP**
Zero-pad [27]	14.80	54.12	71.33	15.95	20.58	68.38	85.79	26.92
TONE [45]	12.52	50.72	68.60	14.42	20.82	68.86	84.46	26.38
HCML [45]	14.32	53.16	69.17	16.16	24.52	73.25	86.73	30.08
cmGAN [47]	26.97	67.51	80.56	27.80	31.63	77.23	89.18	42.19
HSME [50]	20.68	62.74	77.95	23.12	-	-	-	-
BDTR [52]	27.32	66.96	81.07	27.32	31.92	77.18	89.28	41.86
eBDTR [52]	27.82	67.34	81.34	28.42	32.46	77.42	89.62	42.46
D${}^{2}$RL [53]	28.90	70.60	82.40	29.20	-	-	-	-
MSR [44]	37.35	83.40	93.34	38.11	39.64	89.29	97.66	50.88
AlignGAN [48]	42.40	85.00	93.70	40.70	45.90	87.60	94.40	54.30
JSIA-ReID [49]	38.10	80.70	89.90	36.90	43.80	86.20	94.20	52.90
Xmodal [54]	49.92	89.79	95.96	50.73				
MACE [10]	51.64	87.25	94.44	50.11	57.35	93.02	97.47	64.79
DDAG [16]	54.75	90.39	95.81	53.02	61.02	94.06	98.41	67.98
HAT [24]	55.29	92.14	97.36	53.89	62.10	95.75	99.20	69.37
TSLFN + HC [21]	56.96	91.50	96.82	54.95	59.74	92.07	96.22	64.91
DAPR [28]	46.00	87.90	96.00	43.90	46.20	89.2.00	96.70	55.80
WIT [22]	59.20	91.70	96.50	57.30	60.70	94.10	98.40	67.10
AGW [46]	47.50	84.39	92.14	47.65	54.17	91.14	95.98	62.97
FBP-AL [55]	54.14	86.04	93.03	50.20	-	-	-	-
CMAlign [7]	55.41	-	-	54.14	58.46	-	-	66.33
NFS [6]	56.91	91.34	96.52	55.45	62.79	96.53	99.07	69.79
CPN [56]	42.48	87.12	95.62	44.90	-	-	-	-
KSD [51]	61.07	93.15	97.86	58.76	64.09	95.78	98.89	70.57
Ours	**61.89**	**93.56**	**97.86**	**60.12**	**64.24**	**96.58**	**99.08**	**71.04**

**Table 2:** Comparisons with the state-of-the-art methods on RegDB for visible-infrared and infrared-visible settings. R-i indicates Rank-i. The bold indicates the best result.

Setting	V-T				T-V			
**Methods**	**R-1**	**R-10**	**R-20**	**mAP**	**R-1**	**R-10**	**R-20**	**mAP**
Zero-pad [27]	17.74	34.21	44.35	18.90	16.63	34.68	44.25	17.82
HCML [45]	24.44	47.53	56.78	20.08	21.70	45.02	55.58	22.24
BDTR [52]	33.56	58.61	67.43	32.76	32.92	58.46	68.43	31.96
eBDTR [52]	34.62	58.96	68.72	33.46	34.21	58.74	68.64	32.49
D${}^{2}$RL [53]	43.40	66.10	76.30	44.10	-	-	-	-
MSR [44]	48.43	70.32	79.95	48.67	-	-	-	-
HSME [50]	50.85	73.36	81.66	47.00	50.15	72.40	81.07	46.16
AlignGAN [48]	57.90	-	-	53.60	-	-	-	-
JSIA-ReID [49]	48.50	-	-	49.30	48.10	-	-	48.90
Xmodal [54]	62.21	83.13	91.72	60.18	-	-	-	-
DDAG [16]	69.34	86.14	91.49	63.46	68.06	85.15	90.31	61.80
HAT [24]	71.83	87.16	92.16	67.56	70.02	86.45	91.61	66.30
MACE [10]	72.37	88.40	93.59	69.09	72.12	88.07	93.07	68.57
DAPR [28]	61.50	81.60	88.70	59.40	-	-	-	-
AGW [46]	70.10	-	-	66.40	-	-	-	-
FBP-AL [55]	73.98	89.71	93.69	58.24	70.05	89.22	93.88	66.61
WIT [22]	85.00	96.90	98.80	75.90	-	-	-	-
CMAlign [7]	74.17	-	-	67.64	72.43	-	-	65.46
NFS [6]	80.54	91.96	95.07	72.10	77.95	90.45	93.62	69.79
CPN [56]	51.29	71.15	79.79	49.37	-	-	-	-
KSD [51]	76.66	90.19	93.84	69.63	73.64	89.22	93.10	67.41
Ours	**89.37**	**97.62**	**99.08**	**78.74**	**84.51**	**95.83**	**98.01**	**73.75**

### 4.5. Ablation Studies

We conduct ablation experiments on SYSU-MM01 with the all-search set-up to assess the effectiveness of each key component of our method. The detailed results are presented in Table 3. *B* represents the baseline which is implemented by the Local Feature Extractor and optimized by the HC loss and the cross-entropy loss. GAT indicates that the node of the graph is built by the single local feature of the pedestrian image, and LPGAT-*k* is the LPGAT module without using the contextual attention coefficient.

From Table 3, several conclusions can be drawn as follows. First, we observe that *B* + GAT exceeds *B* with 0.7% in Rank-1 accuracy and 1.08% in mAP accuracy, respectively. It indicates that aggregating the local features of different modalities can improve the discrimination of features. Second, *B* + LPGAT-*k* improves the performance compared with *B* + GAT, which demonstrates the effectiveness of using the paired local features from different modalities to build a graph. Third, the comparison between *B* + LPGAT and *B* + LPGAT-*k* proves the effectiveness of the contextual attention coefficient which is beneficial to obtain accurate information propagation. Fourth, *B* + $C^{3}L$ outperforms *B* by 4.46% in Rank-1 accuracy and 3.67% in mAP accuracy, respectively. The proposed $C^{3}L$ could facilitate the deep model to learn the completed distance metric by constraining the distance between local features and their heterogeneous centers to narrow the gap of different modalities. Finally, the performance is further improved when combining LPGAT and $C^{3}L$, which demonstrates they could mutually reinforce.

### 4.6. Parameters Analysis

There are several key parameters in the proposed method. We evaluated the effect of different parameter values on all-search mode in SYSU-MM01, and the experimental results can be generalized to other cross-modality person ReID settings.

**Figure 4:**
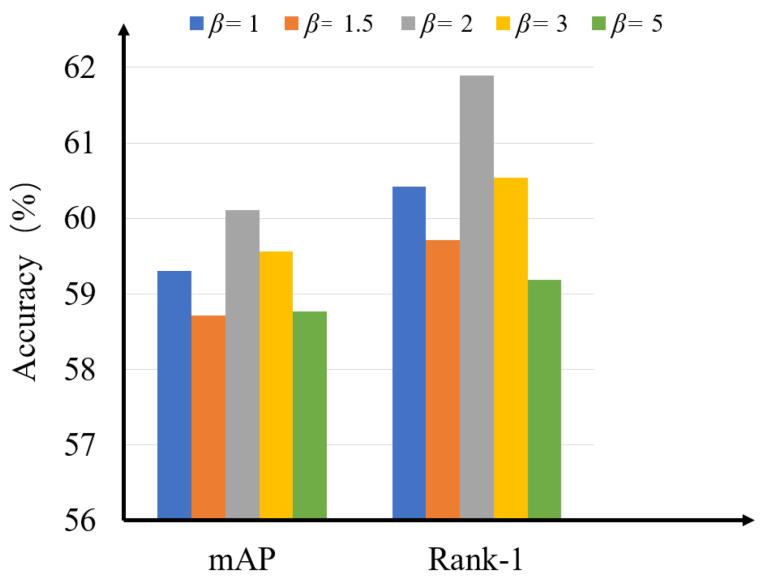
The influence of the hyperparameter β in mAP accuracy and Rank-1 accuracy.

**The impact of the hyperparameter β.** We perform the experiments with different values of β in Equation (4) to evaluate the performance of the proposed method which is shown in Figure 4. From the figure, it can be seen that the performance peaks at β = 2, and it drops as β increases. Therefore, we set β to 2.

**Figure 5:**
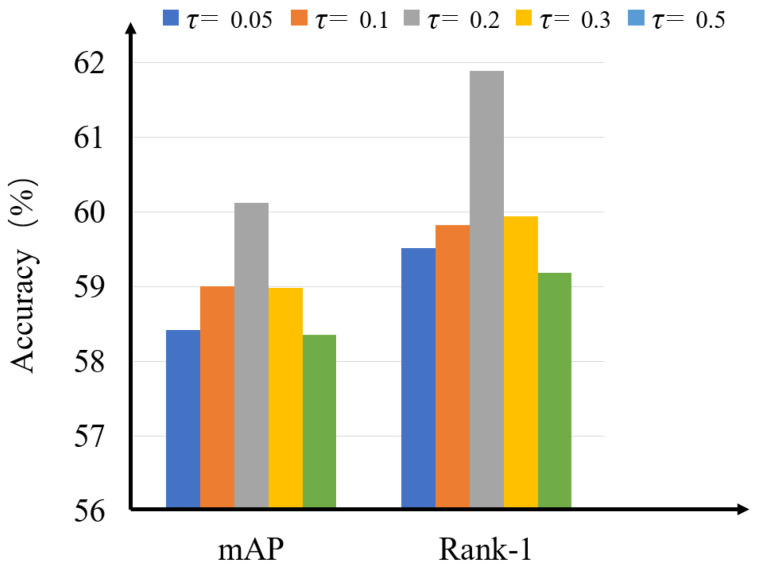
The influence of the scalar temperature parameter τ in Rank-1 accuracy and mAP accuracy.

**The impact of the scalar temperature parameter τ.** The scalar temperature parameter τ is an important parameter that controls the range of similarity between the local features and their heterogeneous centers in Equations (8) and (10). The experimental results with different values of τ are shown in Figure 5 where the performance becomes better as τ increases and the performance drops when τ>0.2. Hence, the optimal value of τ is 0.2.

**Figure 6:**
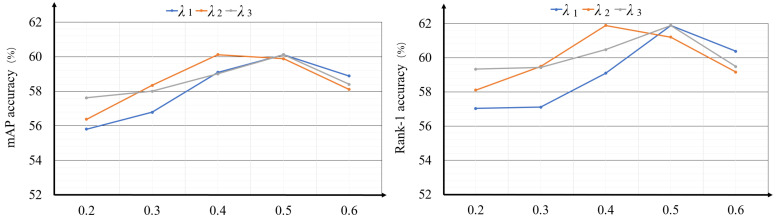
The influence of the trade-off parameters $\lambda_{1}$, $\lambda_{2}$ and $\lambda_{3}$ in mAP accuracy and Rank-1 accuracy.

**The impact of the trade-off parameters $\lambda_{1}$, $\lambda_{2}$ and $\lambda_{3}$.** The trade-off parameters $\lambda_{1}$, $\lambda_{2}$ and $\lambda_{3}$ in Equation (12) control the importance of different losses. To search the optimal values of $\lambda_{1}$, $\lambda_{2}$ and $\lambda_{3}$, we experimentally test different value combinations of them, and to conveniently display, we fix two parameters with the optimal values and show the influence of the other parameter. The results are shown in Figure 6 where we can see that when $\lambda_{1}$ = 0.5, $\lambda_{2}$ = 0.4 and $\lambda_{3}$ = 0.5 the performance is best.

## 5. Visualization

**Figure 7:**
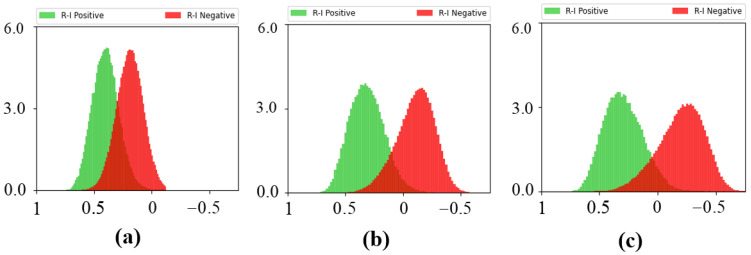
The distributions of the cosine similarity scores of positive and negative pairs of (**a**) *B*, (**b**) *B* + LPGAT and (**c**) *B* + $C^{3}L$. The *x* axis shows the cosine similarity scores between RGB images and IR images, and the *y* axis shows the frequency statistics of the cosine similarity score.

To intuitively verify the effectiveness of our method, we visualize the cosine similarity distribution of cross-modality positive and negative pairs (R-I positive and R-I negative) of *B*, *B* + LPGAT and *B* + $C^{3}L$ as shown in Figure 7. From the figure, we can see that the distribution of *B* + LPGAT and *B* + $C^{3}L$ are more separate than that of *B*. It demonstrates that LPGAT and $C^{3}L$ could improve the discrimination of features for cross-modality person ReID.

## 6. Conclusions

In this paper, we presented LPGAT for cross-modality person ReID to model the correlation between paired local features derived from distinct modalities. Meanwhile, we propose the contextual attention coefficient to ensure accurate information propagation on the graph. In addition, we propose $C^{3}L$ to decrease the modality gap for cross-modality person ReID by constraining the distance between local features and their heterogeneous centers. The results of experiments on two commonly used datasets demonstrate that the proposed approach surpasses the state-of-the-art approaches. In future work, we will extend our approach to video sequences for the cross-modal ReID domain. Considering that in practical ReID application scenarios, multiple tasks such as pedestrian attribute recognition and pose estimation often need to be performed simultaneously, the joint learning of multiple tasks will be considered in the future to make full use of multimodal information and improve the performance of pedestrian re-identification.

## Figures and Tables

**Table 3:** Ablation studies on SYSU-MM01 on the all-search setting. The bold indicates the best result.

Methods	R-1	R-10	R-20	mAP
*B*	56.26	91.41	96.54	55.16
*B* + GAT	56.96	91.89	96.75	56.24
*B* + LPGAT-*k*	58.01	92.83	97.67	56.63
*B* + LPGAT	59.07	93.25	97.68	57.42
*B* + $C^{3}L$	60.72	93.53	97.83	58.83
Ours (*B* + LPGAT + $C^{3}L$)	**61.89**	**93.56**	**97.86**	**60.12**

## Data Availability

All datasets used for training and evaluating the performance of our proposed approach are publicly available and can be accessed from [27,43].
